# The psychological impact of displacement and female genital mutilation/cutting

**DOI:** 10.7189/jogh.15.03011

**Published:** 2025-03-21

**Authors:** Sargun Kaur Virk, Andrew Robert Milewski, Leslie Bull, Gunisha Kaur

**Affiliations:** 1 Department of Anesthesiology, Weill Cornell Medicine, York Avenue, New York, New York, USA; 2 Department of Medicine, University of California, Los Angeles, Hilgard Avenue, Los Angeles, California, USA

## Abstract

Although 230 million people worldwide have undergone female genital mutilation/cutting (FGM/C), its psychological consequences remain understudied. Asylum-seekers may face unique biopsychosocial burdens when migrating to countries where FGM/C is not a cultural norm. We conducted a retrospective observational study of 50 asylum seekers evaluated at the Weill Cornell Center for Human Rights between 2010 and 2020 to characterize the psychological sequelae of FGM/C. Psychological symptoms were reported in 86% of cases, with anxiety, depressed/sad mood, aversion to sexual activity and nightmare being the most common. Formal psychological diagnoses were made for 32% of cases with 30% diagnosed with posttraumatic stress disorder, 20% with major depressive disorder, and 6% with generalised anxiety disorder. Additionally, 74% of cases had experienced other forms of trauma(s), including domestic violence, sexual violence, and kidnapping signaling that violence experienced in this population is complex. Psychological disorders were diagnosed in 93% of individuals who underwent a psychological evaluation, versus 9% of those who did not, despite similar trauma history. There is a role for psychological evaluation and symptom screening for asylum-seekers who have undergone FGM/C.

Forced displacement has sharply increased over the past decade, with nearly 120 million people displaced worldwide, half of whom are women [1]. Displaced women face a high risk of gender-based violence (GBV), with over 70% experiencing it in some displacement contexts [2]. Beyond the immediate physical dangers, GBV leads to long-term psychological trauma, making displaced women more likely to develop mental health disorders such as posttraumatic stress disorder, depression, and anxiety compared to non-displaced populations [3].

The vulnerability of displaced women often begins in their home countries, where cultural norms may limit their access to education and economic independence, reinforcing their dependence on men. Conflict and instability amplify these disadvantages, increasing discrimination and the risk of GBV throughout the migration journey [4]. As critical safety nets collapse, women’s rights are eroded by conflict, forced displacement, family separation, and the breakdown of community and institutional protections. While causation has not been fully established, reports indicate a rise in GBV following mass displacement [5,6].

A type of GBV is female genital mutilation/cutting (FGM/C), which involves the partial or total removal of the external female genitalia for non-medical reasons and affects over 230 million children and adults worldwide [7]. Around 40% of girls who undergo FGM/C live in countries affected by conflict or instability, and its prevalence can reach up to 80% among certain populations of female asylum seekers [7,8]. As they move from a country where FGM/C is a cultural norm to one in which it is considered a violation of human rights, displaced women may experience isolation, shame, and a lack of understanding from healthcare providers [9,10]. As a result, migrants and asylum seekers who have undergone FGM/C often experience a double burden of psychological trauma from the procedure itself and discrimination in their post-migration communities. These layered traumas may intensify the psychological effects of FGM/C. While extensive research has explored the medical consequences of FGM/C, including dyspareunia, urinary tract infection, long-term obstetric complications, and chronic physical pain, its social, economic, and psychological impacts remain underexplored [11,12]. A limited number of studies have linked FGM/C to increased rates of anxiety, depression, and post-traumatic stress disorder [12].

FGM/C is rarely an isolated event; rather, it may be a part of a broader, lifelong pattern of GBV [13]. Women who have undergone FGM/C often face multiple, compounding forms of violence, including intimate partner violence, sexual violence, and human trafficking [14–17]. These intersecting forms of trauma and violence significantly increase their risk of developing long-term mental, physical, and emotional health issues [15]. Nevertheless, the concept of polyvictimisation – the overlapping risks of war-related, gender-based, and racially motivated violence – has rarely been used to understand the impact of FGM/C and its relationship to other forms of violence [6].

To better understand the psychological impact of FGM/C on displaced women, we conducted a retrospective study of 50 asylum applicants who had undergone FGM/C and who were evaluated at the Weill Cornell Center for Human Rights between 2010 and 2020. These applicants underwent forensic psychological, medical, and gynaecological evaluation, either individually or in combination. Findings from these assessments were documented in medical-legal affidavits. We aimed to identify and characterise the psychological symptoms, diagnoses, and additional traumas associated with FGM/C. We defined psychological symptoms using the American Psychological Association’s list of signs and symptoms of mental illness and the Hopkins Symptom Checklist – 25 [18,19]. We recorded psychological diagnoses as documented by the evaluating clinician, and the clinical intake form provided the list of traumas [20].

Our analysis showed that 86% of the asylum applicants who had undergone FGM/C had at least one psychological symptom. The most frequently reported symptoms were anxiety (34%), depressed/sad mood (34%), aversion to sexual activity (28%), and nightmares (28%). Formal psychological diagnoses were made for 32% of the applicants, with posttraumatic stress disorder (30%), major depressive disorder (20%), and generalised anxiety disorder (6%) being the most common. Additionally, 74% of the women in our sample had experienced at least one additional trauma, most often domestic violence (58%), sexual violence (50%), kidnapping (16%), or violence perpetrated by government officials (8%).

Among the 15 applicants who received a psychological evaluation, 14 (93%) were diagnosed with a psychological disorder. The odds of receiving a psychological diagnosis were more than 100 times higher when a psychological evaluation was performed (odds ratio = 119.3; 95% confidence interval = 12.36–149.8) compared to cases without such evaluation. These findings highlight the significant psychological burden within this population, which remains largely underdiagnosed. In addition to standard medical and gynaecological assessments, improved protocols for identifying and treating psychological conditions in individuals affected by FGM/C are crucial. Given the high prevalence of sexual and domestic violence in this population, screening for intimate partner violence is also warranted.

The application of trauma-informed care principles in treating FGM/C survivors has been previously recommended [11]. This approach acknowledges that perceptions of FGM/C vary across cultural contexts and emphasises the importance of ‘meeting patients where they are’. While FGM/C is often a traumatic experience, many individuals who have undergone the procedure continue to support the practice due to deeply ingrained cultural beliefs. Additionally, individual views on FGM/C may shift over time, particularly with exposure to new cultural environments [10]. Integrating trauma-informed care into clinical practice could enhance patient trust in providers and increase their willingness to engage in healthcare.

Refugees and asylum seekers often face barriers to accessing healthcare, including language barriers, lack of information, transportation costs, logistical challenges, and limited opportunities for shared decision-making [21,22]. Addressing these challenges requires a community-based approach that adopts a participatory, localised, and bottom-up strategy to meet the unique needs of this population [23]. The incorporation of trauma-informed care alongside community engagement is essential for effectively integrating these individuals into the healthcare system.

Nations worldwide should develop models similar to the UK's National Health Service FGM enhanced data set for comprehensive data collection, which can help identify individuals at risk of FGM/C and enable tailored services to reach those in need [24]. The adoption of national FGM/C community-based support clinics can ensure comprehensive care while collaborating with communities to prevent future cases through education about the medical, psychological, and legal consequences of FGM/C [25]. Approaching FGM/C in asylum seekers through an intersectional lens will promote the development of accessible, culturally sensitive social support systems, ensuring that comprehensive care is available. Insights from this study highlight the importance of a holistic approach to healthcare that addresses the complex challenges faced by forcibly displaced women who have undergone FGM/C. By implementing targeted interventions and culturally informed care, we can better support the health and well-being of this vulnerable population.
